# Correction: REV-ERB regulates RORγt^+^ regulatory T cell specification and function through the Bhlhe40-c-Maf axis

**DOI:** 10.1084/jem.2024146312152025C

**Published:** 2025-12-18

**Authors:** Xianting Hu, Zhi Liu, Yao Li, Yannan You, Kaiye Yue, Yuqiong Liang, Chin-San Loo, Jingting Yu, Matthias Leblanc, Dehui Wang, Huabin Li, Ye Zheng

Vol. 223, No. 2 | https://doi.org/10.1084/jem.20241463 | December 8, 2025

The authors regret that there were errors in the originally published version of their article. In Fig. 5, panels D and E have been switched to match the order of description in the main text. Panel D shows flow cytometric analysis of RORγt expression in Foxp3^+^ iTreg cells with ectopic expression of Bhlhe40 or control vector, while panel E shows flow cytometric analysis of RORγt expression in Foxp3^+^ iTreg cells transduced with sgNT and sgBhlhe40. In addition, “*Il1r1* (encoding IL33R)” was corrected to “*Il1r****l****1* (encoding IL33R)” in the “REV-ERB regulates the expression of core signature genes . . .” section of the Results. The corrected text and the original and corrected figures and legends are shown here, with changes indicated in bold and underlined. These errors do not affect the conclusions of the study. The errors appear in PDFs downloaded before December 15, 2025.

## Results

### **REV-ERB regulates the expression of core signature genes in colonic RORγt** ^**+**^ **Foxp3** ^**+**^ **Treg cells** fv*(first paragraph)*

To investigate how REV-ERB regulates the transcriptional programs in Treg cells, we performed RNA-seq analysis of colonic Treg cells isolated from WT and REV-ERB cKO mice after TNBS challenge. We identified 1030 upregulated genes and 952 downregulated genes (false discovery rate [FDR] <0.05) in REV-ERB–deficient Treg cells compared with WT controls (Fig. 3 A). REV-ERB–deficient colonic Treg cells expressed a lower level of Treg core genes including *Foxp3*, *Ctla4*, *Icos*, *Gzmb*, *Il10*, *Lag3*, *Nt5e* (encoding CD73), *Entpd1* (encoding CD39), ***Il1rl1* (encoding IL33R)**, *Lrrc32* (encoding GARP), and *Itgae* (encoding CD103). Furthermore, the expression of genes encoding RORγt and c-Maf, a transcription factor critical for normal RORγt^+^Foxp3^+^ Treg cell function and differentiation, was also reduced in REV-ERB–deficient Treg cells (Fig. 3 A). Previous studies showed that although they are generally stable and exert enhanced suppressive function, colonic RORγt^+^Foxp3^+^ Treg cells could be proinflammatory in individuals with IBD (Quandt et al., 2021). In agreement with these observations, we found that the genes encoding proinflammatory cytokines, including *Il17a*, *Il17f*, *Il4*, and *Il5*, were significantly upregulated in REV-ERB–deficient colonic Treg cells (Fig. 3 A). To assess whether REV-ERB has a global impact on colonic Treg signature gene expression, we performed gene set enrichment analysis (GSEA) using a published gene set of 364 colonic Treg signature transcripts identified previously (Sefik et al., 2015). Indeed, these colonic Treg signature transcripts were significantly enriched in WT colonic Treg cells than REV-ERB–deficient colonic Treg cells (Fig. 3 B). Next, we extended to examine whether REV-ERB regulates RORγt-dependent transcripts in colonic Treg cells. Again, GSEA revealed that RORγt-dependent signature genes were preferably enriched in WT colonic Treg cells (Fig. 3 B), suggesting that REV-ERB is required for colonic RORγt^+^Foxp3^+^ Treg signature gene expression.

**Figure 5.**
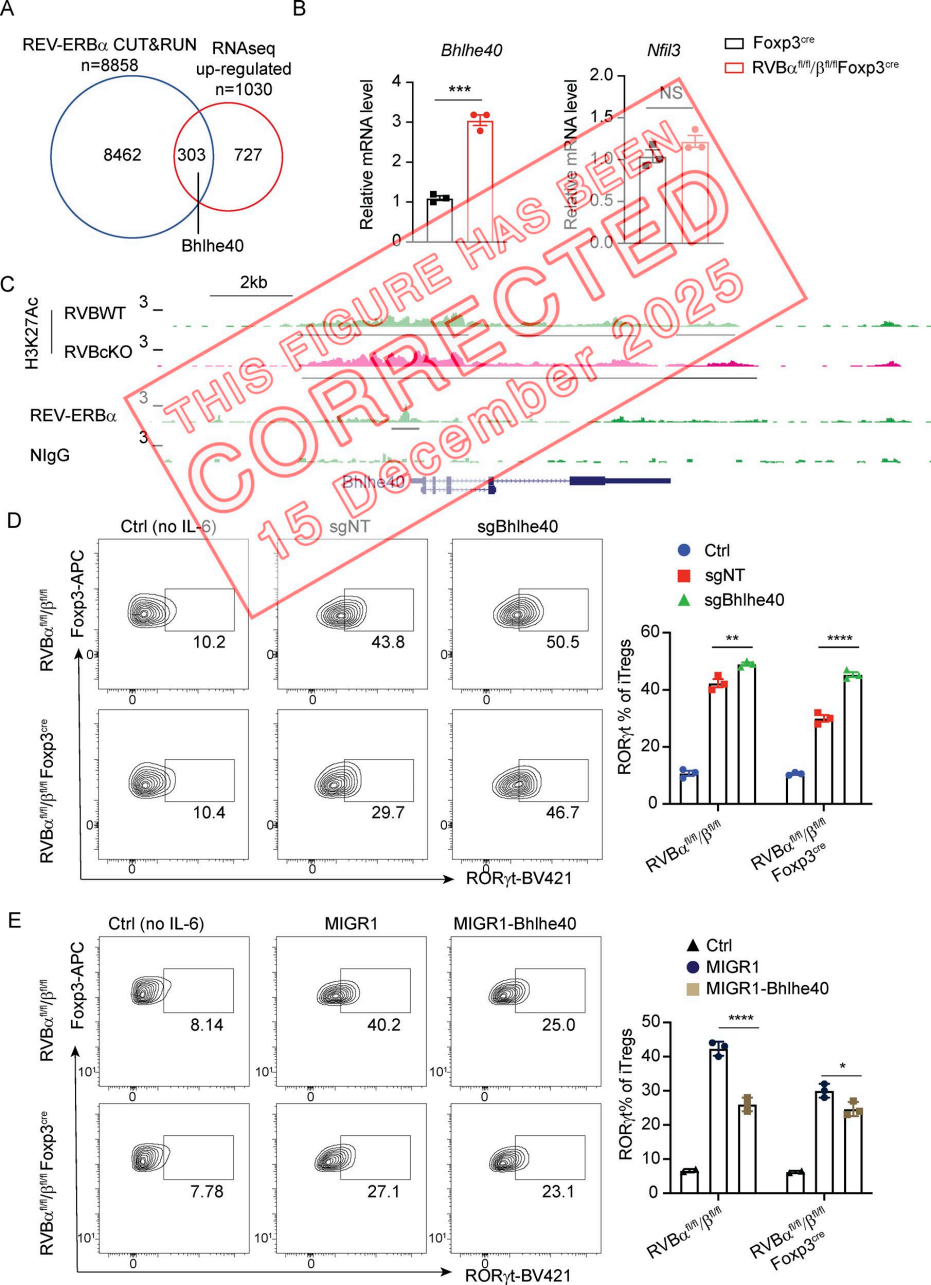
**Bhlhe40 is a critical repressor downstream of REV-ERB that regulates RORγt** ^**+**^ **expression in Treg cells. (A)** Venn diagram of overlapping REV-ERBα–bound genes from CUT&RUN and genes with upregulated expression in REV-ERB–deficient Treg cells from RNA-seq. **(B)** RT-qPCR analysis of *Bhlhe40* and *Nfil3* expression in WT and REV-ERB–deficient Treg cells. ***P = 0.0002. Data are representative of two independent experiments. Statistical significance was determined using Student’s *t* test analysis. **(C)** CUT&RUN tracks showing H3K27Ac- and REV-ERBα–bound peaks at the *Bhlhe40* locus, with underlines detected as peaks by HOMER. **(D)** Flow cytometric analysis of RORγt expression in Foxp3^+^ iTreg cells transduced with sgNT and sgBhlhe40. **P = 0.0094, ****P < 0.0001. **(E)** Flow cytometric analysis of RORγt expression in Foxp3^+^ iTreg cells with ectopic expression of Bhlhe40 or control vector (*n* = 3 biologically independent replicates per group). ****P < 0.0001, *P = 0.0131. Data in D and E are representative of at least two independent experiments. Statistical significance was determined using two-way ANOVA.

**Figure 5.**
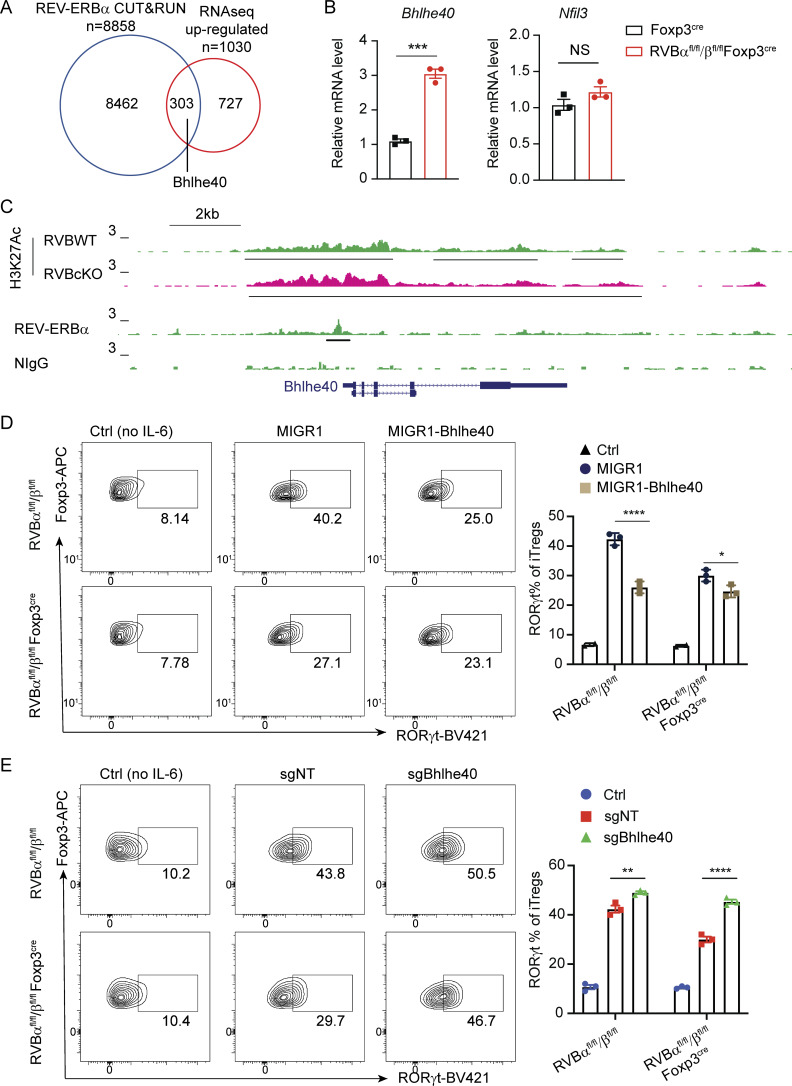
**Bhlhe40 is a critical repressor downstream of REV-ERB that regulates RORγt** ^**+**^ **expression in Treg cells. (A)** Venn diagram of overlapping REV-ERBα–bound genes from CUT&RUN and genes with upregulated expression in REV-ERB–deficient Treg cells from RNA-seq. **(B)** RT-qPCR analysis of *Bhlhe40* and *Nfil3* expression in WT and REV-ERB–deficient Treg cells. ***P = 0.0002. Data are representative of two independent experiments. Statistical significance was determined using Student’s *t* test analysis. **(C)** CUT&RUN tracks showing H3K27Ac- and REV-ERBα–bound peaks at the *Bhlhe40* locus, with underlines detected as peaks by HOMER. **(D) Flow cytometric analysis of RORγt expression in Foxp3**^**+**^iTreg cells with ectopic expression of Bhlhe40 or control vector (*n* = 3 biologically independent replicates per group). ****P < 0.0001, *P = 0.0131. (E) Flow cytometric analysis of RORγt expression in Foxp3^**+**^iTreg cells transduced with sgNT and sgBhlhe40. **P = 0.0094, ****P < 0.0001. Data in D and E are representative of at least two independent experiments. Statistical significance was determined using two-way ANOVA.

